# Effect of the Novel Coronavirus Pneumonia Pandemic on Medical Students’ Psychological Stress and Its Influencing Factors

**DOI:** 10.3389/fpsyg.2020.548506

**Published:** 2020-10-14

**Authors:** Wan Ye, Xinxin Ye, Yuanyuan Liu, Qixi Liu, Somayeh Vafaei, Yuzhen Gao, Huiqin Yu, Yanxia Zhong, Chenju Zhan

**Affiliations:** ^1^Department of Nursing, Xiamen Medical College, Xiamen, China; ^2^School of Public Health, Zhejiang University School of Medicine, Hangzhou, China; ^3^Department of Nursing, Nanjing Hospital of Chinese Medicine Affiliated to Nangjing University of Chinese Medicine, Nanjing, China; ^4^Department of Nursing, Mindong Hospital of Fujian Medical University, Fuan, China; ^5^Department of Molecular Medicine, Faculty of Advanced Technologies in Medicine, Iran University of Medical Sciences, Tehran, Iran; ^6^Department of Clinical Laboratory, Sir Run Run Shaw Hospital, Zhejiang University School of Medicine, Hangzhou, China; ^7^Department of Nursing, Shijiazhuang Medical College, Shijiazhuang, China

**Keywords:** novel coronavirus pneumonia, medical students, stress, perceived stress scales, influencing factors

## Abstract

In December 2019, an outbreak of the novel coronavirus pneumonia infection occurred in Wuhan City, Hubei Province, China, and it has received substantial attention globally. Few studies have investigated the psychological stress of students in Health University during the COVID-19 outbreak, and almost no work has attended to the influencing factors that may cause their psychological stress risk. This cross-sectional, survey-based, region-stratified study collected demographic data and mental measurement from 2,498 medical students and 1,177 non-medical students in 31 provinces from March 5, 2020, to March 10, 2020, in China. The psychological stress was measured using the Chinese Perceived Stress Scales (CPSS) under a self-design questionnaire. Sociodemographic, major characteristics, and knowledge of the novel coronavirus pneumonia were also identified as potential influencing factors of stress. The study revealed that medical students are suffering from more stress than non-medical students almost in all provinces of China. Four influencing factors including level of familiarity with the novel coronavirus, family income, major of students, and status of the intern student can be significantly related to students’ stress in the medical group by using the univariate and multivariate analysis. Further analysis showed that students with low stress had a greater number of positive psychological emotions and a lower number of negative psychological emotions than with medical students with high stress. In addition, high stress caused low enthusiasm for learning in these medical students and lead to little/no willingness to do professional medical work in the future. In conclusion, we need to increase the level of our knowledge related to the novel coronavirus pneumonia to reduce stress and strongly focus on the special populations in medical students with certain features, such as intern students, clinical nursing students, and low-income families, to improve their learning attitudes and establish positive professional mental outlooks.

## Introduction

In December 2019, an outbreak of the novel coronavirus pneumonia infection occurred in Wuhan City, Hubei Province, China, and has spread to the rest of the country. This speedy onset crisis was accompanied with strong infectivity, rapid rate of course changes of the disease, and the general susceptibility of the population. On January 20, 2020, the National Health Committee included pneumonia caused by the new coronavirus infection as a Class B infectious disease under Category A of Infectious Disease Management (Drosten et al., 2003; Zaki et al., 2012; Chang et al., 2020). The World Health Organization 2020 has identified the novel coronavirus infection as a Public Health Emergency of International Concern and named it “COVID-19” (World Health Organization [WHO], 2020). As of March 6, 2020, more than 3000 medical staff in Hubei Province have been infected with COVID-19, of which 40 and 60% have been infected in hospitals and communities, respectively. All of them were local medical staff in Hubei Province, and most of them specialize in non-communicable diseases (The State Council Information Office of the People’s Republic of China, 2020). As of 19:00 on March 25, 2020, China had confirmed 81,896 cases of COVID-19, including 3287 dead and 73,793 recovered (National Health Commission of the People’s Republic of China, 2020).

From January 25, 2020, 30 provincial-level administrative units have initiated major public health emergency level responses for effective prevention and control (Li Z. et al., 2020). On January 27, 2020, the Minister of Education issued a notice on the extension of the spring semester (Ministry of Education of the People’s Republic of China, 2020). Colleges and universities in each region have to start in accordance with the control of the local epidemic and the unified deployment of the local education administration and government (Rothe et al., 2020). Many college students would be required to take home isolation because of the implementation of strict traffic control and the postponement of the opening of colleges. However, these young students in the learning stage are still in the mature period of psychological development. In the face of such a ferocious epidemic and heavy academic work, they will be burdened by the pressure caused by COVID-19 or social isolation and interruption of normal school activities. In addition, cyberspace rumors can exacerbate psychological stress on students due to being unfamiliar with the novel disease. The widespread news also causes more concerns related to the severity of the disease. Lazarus and Folkman showed that when someone has to face huge hazards that are beyond their ability to handle, the physical and mental health are affected directly (Liu et al., 2015). As the first mental problem, stress can change students’ feelings through further physical and mental symptoms (Yuan and Lin, 2009). Therefore, focusing on the particularity of stress is helpful to improve the ability of early prevention of mental illness.

Among these students, medical students are considered a special population. Although the COVID-19 epidemic is very dangerous, there are still many other factors that would affect students’ psychological stress. Firstly, during the outbreak response, online teaching approaches have been launched successively to avoid delays in study progress. But the lack of interaction and teaching materials and the unfamiliar classroom environment increase the concerns and discomfort among the students (Yang et al., 2020). Secondly, based on expectations for the future, medical students may suffer more stress than other professional students in academic and employment in the current environment (McGuire, 1966; Dyrbye and Shanafelt, 2011; Dyrbye et al., 2011; Voltmer et al., 2012). Furthermore, some medical interns were even required to be on the frontline to fight the COVID-19 in some cities of China. The poor situation of frontline medical workers has attracted much more attention from medical students, which furtherly enhance the stress perception of medical students. Additionally, a lot of basal characteristics of personal students can also change the personal psychological stress, such as family incoming, student’s age and gender, etc. For these factors, seeking the sources of students’ stress with a great importance are worth exploring.

As we know, psychological stress can effect the overall mental health of these medical students (Al-Rabiaah et al., 2020), such as anxiety and depression, etc. (Cao et al., 2020; Liu J. N. et al., 2020). This outbreak has highlighted the fragility of mental resilience. For now, there are many researches focusing on the mental health of medical care workers who were exposed to COVID-19 with increasing frequencies of mental symptoms (Liu C. Y. et al., 2020). The rates of anxiety and depression among medical students are almost 12.5–23.48% (Chen et al., 2014; Zhao et al., 2018) and 13.2–48.7% (Chi et al., 2019; Xiong et al., 2019), respectively. Meanwhile, the researchers also show that the numbers of psychological mental health are generally increasing among college students (Cao et al., 2020). Therefore, to explore the correlation analysis between these mental health symptoms in medical students would bring huge benefits to the comprehensive management of students in the pandemic period.

Of note, if high psychological stress and poor employment environment both were part of a vicious circle, it may lead to the decline of academic and employment performance and obviously reduce the quality of life of medical students (Dyrbye et al., 2005). To clarify, if the perceived stress of medical students has existed for a long time, it would directly affect the choice of medical career (McGuire, 1966; Kumar et al., 2019). In extreme cases, mental illness of these students during this period may even lead to students’ suicide (Singh et al., 2016; Kumar et al., 2019; Li H. Y. et al., 2020).

Despite the importance of these issues, only a few studies have investigated the psychological stress of medical students in Health University in the COVID-19 outbreak in China. Therefore, this study’s aim is to design an online survey based on the CPSS questionnaire by collecting the demographic data (such as gender, age, educational background, nationality, monthly income of families and understanding of the disease, etc.) to describe the distribution of the psychological stress of medical students and to identify its influencing factors in China during the pandemic period. In addition, we also focus on exploring the relationship between stresses with psychologies phenomena and the changing of attitudes of learning and employment. Our findings might help governments, schools, or health authorities to recognize the causes of increased stress and their influences in medical students, and then to provide early effective measures to reduce that stress.

## Materials and Methods

### Study Population

Using the cross-sectional research method, online students were investigated anonymously online by snowballing through questionnaires sent to WeChat and QQ. We restricted the IP address of each device (mobile phone, computer, and tablet) to answer the survey only once. The survey period is from March 5, 2020, to March 10, 2020. The questionnaire survey platform was developed by Changsha Ranxing Information Technology Co., Ltd. In total, 3,680 questionnaires were distributed and 3,675 valid questionnaires were recovered, with an effective rate of 99%. The inclusion criteria of this study were as follows: (1) Full-time college students aged 16∼31 years old; (2) Be able to read and write; (3) Those who are willing to participate in this project. The exclusion criteria included: (1) Those who were unable to complete the study due to severe visual or hearing impairment, mental disorder, etc.; (2) Those with cognitive dysfunction.

### Pilot Survey

Four medical administrators, four medical students, and four non-medical students were selected to conduct a pre-survey to understand the use of the questionnaires in this study. According to the feedback of the respondents, the questionnaire was modified appropriately.

### Data Collection

Using the online questionnaire preparation method, the questionnaire items are entered one by one, and online release and questionnaire were collected. Before the input of the collected data, the errors were checked, and omissions were made up and the logic checked. Additionally, the questionnaires with obvious logic errors and more missing items were eliminated.

### Questionnaire Contents

The first part included the general situation, such as gender, age, educational background, nationality, average monthly income of family members, learning attitude, and professional attitude. For the education background, we divided full-time college students into medical major and non-medical major. The medical major mainly includes clinical medicine and clinical nursing. Non-medical major mainly includes information management and high-speed rail crew, etc. The second part consisted of the Chinese version of perceived stress scale (CPSS). Previous PSS is widely accepted and used for psychological stress assessment. Cohen et al. (1983) developed the PSS in 1983. The Cronbach coefficient of the scale is 0.78, indicating good reliability and validity. Now, the scale has been sinologized by Yang Yanzhong of Zhejiang University (Yang and Huang, 2003). The CPSS can quickly judge the individual stress state through 14 designed questions. The 4, 5, 6, 7, 9, 10, and 13th questions were reverse items, and the total score range was 0–56. A high score indicates high psychological stress. A total score of ≥25 points is defined as high stress. In the current study, we used the CPSS as the subjective index of psychological stress assessment. The Cronbach coefficient of this questionnaire was 0.827 to show good reliability.

### Statistical Analysis

In this questionnaire, the continuous variables were reported as mean and standard deviation (SD) and compared by using the Student’s *t*-test or ANOVA test in two or more groups. LSD test was used to detect the multi-comparison after ANOVA test. The dichotomous data were presented as frequency (%) and compared by using the chi-square or the Fisher’s exact test in two groups. The distribution of mean CPSS score of the students from each province was also calculated. We firstly compared the CPSS score between the medical and non-medical student groups. Then, univariate analysis methods, such as the Student’s *t*-test and chi-square or Fisher’s exact test, were used to explore the candidate variables that related to the high CPSS score in the medical students. The standard mean difference (SMD) of these candidate variables between the two groups was calculated by the “tableone” package in R. Then, the multivariate logistics regression analysis was performed to determine the independent risk factors of the CPSS score. We presented the results of the multivariate analysis on a forest plot for all the comparative Odds Ratio (OR) values with it 95% confidence interval (CI) of the associations. Lastly, the bar plots of some potentially related variables were also used to analyze the differences between high CPSS and low CPSS groups. The main packages, including “forestplot,” “glm,” “ggolot2,” “maps,” “mapdata,” and “tableone,” were applied to visualize and analyze the results and conclusions. All the reported *P-*values with a significance level of 0.05 were defined based on two-sided tests. All statistical processes were performed in the R software (R Foundation for Statistical Computing, Vienna, Austria, and version 3.6.0).

## Results

### Questionnaires Collection and Study Design

This is a cross-sectional, survey-based, region-stratified study and collected demographic data and CPSS measurement from 3,680 students in 31 provinces from March 5, 2020, to March 10, 2020, in China. Specifically, a total of 3,680 questionnaires were distributed and 3,675 valid questionnaires were recovered to analysis, with an effective recovery rate of 99%. To fully demonstrate the role of stress in medical students during the pandemic period, a comprehensive analysis with a flowchart was designed in Supplementary Figure S1.

### Distribution of CPSS Score of Medical and Non-medical Students in China

**FIGURE 1:**
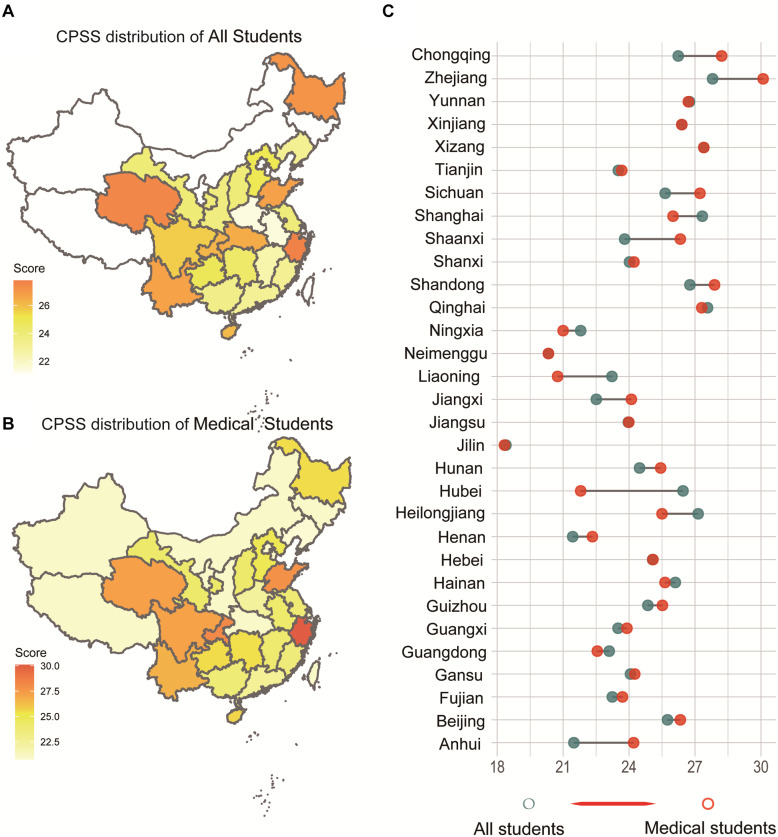
The distribution and difference of the CPSS score of all students in each province of China. **(A)** The distribution of all students. **(B)** The distribution of the CPSS score of medical students. **(C)** CPSS score Changing from all students to medical students in each province of China.

Exactly 68.2% (2,498/3,675) of the students were medical students, whereas the remaining were non-medical students (31.8%, 1,177/3,675) in the questionnaire. First, we presented the distribution of CPSS score of all students in 31 provinces of China (Figure 1A), as well as the medical students (Figure 1B). Apparently, we found a regional heterogeneity in these provinces through the different depth of colors in the distribution map of CPSS score. From Figure 1C, we clearly found an increasing trend of CPSS score in the medical students by comparing with the total students in each Chinese province. The detailed digital results of these figures are included in Supplementary Table S1.

### Demographic Comparison of Medical and Non-medical Students

**FIGURE 2:**
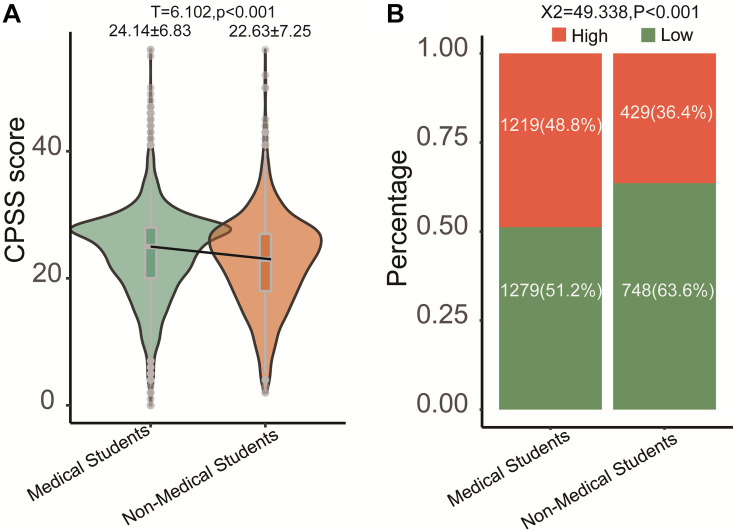
Comparison of medical students with non-medical students. **(A)** Continuous CPSS score; **(B)** category CPSS score (high > 25 vs. low ≤ 25).

To detect the basal demographic characteristics of the medical and non-medical students, we compared collecting variables in Table 1. The differences of some variables, such as sex, age, race, source of the student, and the family income, also were detected between the two groups. By using the *t*-test the gaps between medical and non-medical students in the CPSS score were investigated. We found that the medical students had a higher mean CPSS score than the non-medical students (Figure 2A, CPSS score, medical: 24.14, non-medical: 22.63; *P* < 0.001). We stratified all the students into two groups, namely, the high- and low-CPSS group, by using the cutoff (value = 25) of the CPSS score (Yang and Huang, 2003). Therefore, based on the cut-off of CPSS in our study, the rate of high CPSS was 44.8% (1,648/3,676) in all students. Among them, the rate of medical students with high CPSS was 48.7% (1,219/2,499), and the rate of the students with low CPSS was 36.4% (429/1,178). The bar plot with the chi-square test can also detect the differences between two groups (Figure 2B, medical: 48.8%, non-medical: 36.4%; *P* < 0.001).

**TABLE 1:** The basal characteristics of medical and non-medical students in the design questionnaire.

**Variables**	**Medical students (*n* = 2,498)**	**Non-medical students (*n* = 1,177)**	***P*-value**
Sex (%)			**<0.001**
Male	364(14.6)	350(29.7)	
Female	2,134(85.4)	827(70.3)	
Age [mean (*SD*)]	20.78(1.53)	19.59(1.36)	**<0.001**
Only one child (%)			0.311
No	491(19.7)	249(21.2)	
Yes	2,007(80.3)	928(78.8)	
Race (%)			0.031
Han	2,347(94.0)	1,127(95.8)	
Others	151(6.0)	50(4.2)	
Students source (%)			**<0.001**
City	281(11.2)	194(16.5)	
Town	596(23.9)	268(22.8)	
Rural	1,621(64.9)	715(60.7)	
Income of family (per months)			**<0.001**
–2,000	648(25.9)	209(17.8)	
2,000–3,000	809(32.4)	313(26.6)	
3,001–4,000	429(17.2)	253(21.5)	
4,001–5,000	258(10.3)	193(16.4)	
5,000–	354(14.2)	209(17.8)	
Level of familiar for coronavirus (%)			0.230
Very understanding,	297(11.9)	143(12.1)	
Relatively understanding	1,335(53.4)	588(50.0)	
General understanding	828(33.1)	420(35.7)	
Little understanding	34(1.4)	23(2.0)	
Not at all	4(0.2)	3(0.3)	
Live with family during the period of coronavirus (%)			**0.025**
Yes	2,428(97.2)	1,159(98.5)	
No	70(2.8)	18(1.5)	
Infection cases (%)			0.191
No	2,497(100.0)	1,174(99.7)	
Yes	1(0.0)	3(0.3)	
Number of positive. emotions	3.75(1.34)	3.79(1.29)	0.335
Number of negative emotions	1.98(1.49)	1.86(1.40)	**0.024**
Attitude of learning (%)			**<0.001**
Never	57(2.3)	17(1.4)	
Hardly	131(5.2)	51(4.3)	
Sometimes	1,220(48.8)	508(43.2)	
Often	799(32.0)	423(35.9)	
Always	291(11.6)	178(15.1)	
^#^Intern student (%)			NA
Yes	994(39.8)	NA	
No	1,504(60.2)	NA	
^#^Attitude of medical work (%)			NA
Very willing	1,222(48.9)	NA	
Relatively willing	888(35.5)	NA	
General willing	330(13.2)	NA	
Little willing	44(1.8)	NA	
Unwilling	14(0.6)	NA	

*Note: ^#^Only for medical students; Chi-square test for categorical variables; T-test for continuous variables. Bold font means there is a significant difference between the two groups.*

### Univariate and Multivariate Analysis for High CPSS Score Among Medical Students

**FIGURE 3:**
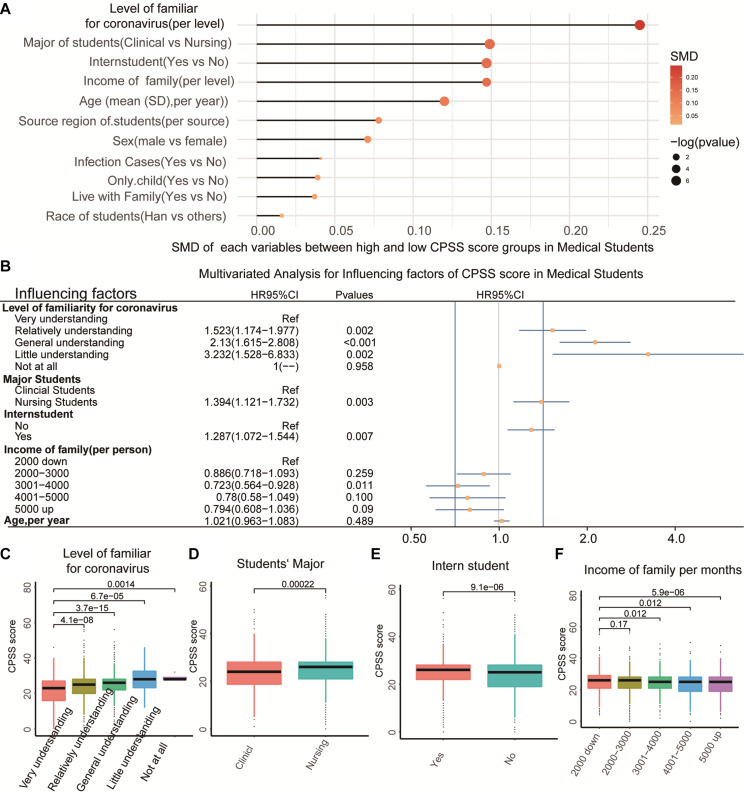
Association of common variables with the CPSS score. **(A)** The SMD value of each variable between high CPSS score and low CPSS score in medical students. **(B)** The multivariable regression for the high CPSS score in medical students. **(C–F)** The significant difference of CPSS scores with four influencing factors.

To select the impact factors that related to the CPSS score among the medical students, we firstly compared the differences in the variables in the medical students with High CPSS score or Low CPSS score. Table 2 showed that five factors (i.e., age, family income, level of familiarity to COVID-19, major of students, and status of the intern student) could be the candidate variables related to the CPSS score in medical students (all *P* < 0.01). Then, we obtained the ranking of the five most relevant factors to CPSS by using the method of SMD in the package of “tableone” in R (Figure 3A). Thereafter, we included the five significantly related variables into multivariate logistic regression analysis. Finally, it showed that four variables were the independent risk factors of the CPSS score in the medical students (Figure 3B, all *p* < 0.05). As a supplement, we also tested the relationship between the continuous CPSS score with these factors by using the *T-*test and the LSD test and obtained results similar to those of the regression and general chi-square tests (Figures 3C–F, *P* < 0.05).

**TABLE 2:** The relationship of influencing factors with CPSS score (high vs. low) in medical students.

**Influencing factors**	**High CPSS score (*n* = 1,219)**	**Low CPSS score (*n* = 1,279)**	***P*-value**
Sex (%)			0.086
Male	162(13.3)	202(15.8)	
Female	1,057(86.7)	1,077(84.2)	
Age [mean (*SD*)]	20.87(1.52)	20.69(1.53)	**0.003**
Only one child (%)			0.359
Yes	230(18.9)	261(20.4)	
No	989(81.1)	1,018(79.6)	
Race (%)			0.761
Han	1,143(93.8)	1,204(94.1)	
others	76(6.2)	75(5.9)	
Source of students (%)			0.153
City	122(10.0)	159(12.4)	
Town	298(24.4)	298(23.3)	
Rural	799(65.5)	822(64.3)	
In come of family (per month)			**0.009**
2,000 down	347(28.5)	301(23.5)	
2,000–3,000	406(33.3)	403(31.5)	
3,001–4,000	190(15.6)	239(18.7)	
4,001–5,000	118(9.7)	140(10.9)	
5,000 up	158(13.0)	196(15.3)	
Level of familiarity for coronavirus (%)			**<0.001**
Very understanding	110(9.0)	187(14.6)	
Relatively understanding	630(51.7)	705(55.1)	
General understanding	453(37.2)	375(29.3)	
Little understanding	22(1.8)	12(0.9)	
Not at all	4(0.3)	0(0.0)	
Live with family (%)			0.418
Yes	1,181(96.9)	1,247(97.5)	
No	38(3.1)	32(2.5)	
Infection cases (%)			0.981
No	1,218(99.9)	1,279(100.0)	
Yes	1(0.1)	0(0.0)	
Major of student (%)			**<0.001**
Clinical	185(15.2)	267(20.9)	
Nursing	1,034(84.8)	1,012(79.1)	
Intern student (%)			**<0.001**
Yes	530(43.5)	464(36.3)	
No	689(56.5)	815(63.7)	

*Note: ^#^Chi square test for categorical variables; T-test for continuous variables; Bold font means there is a significant difference between the two groups.*

### Relationship of CPSS Score With Psychological Phenomena Among Medical Students

**FIGURE 4:**
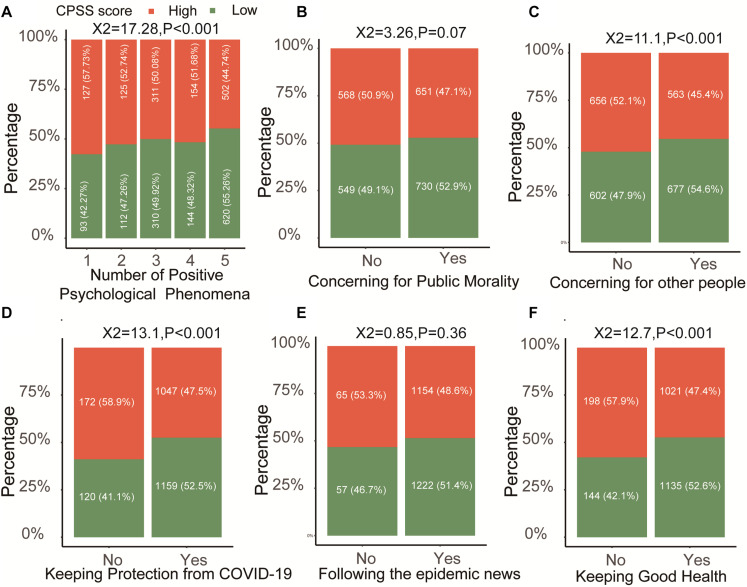
The relationship between CPSS score with different positive psychological phenomena in medical students. **(A)** The trend of the number of positive psychological phenomena in high or low CPSS score groups. **(B–F)** The individual positive psychological phenomenon.

**FIGURE 5:**
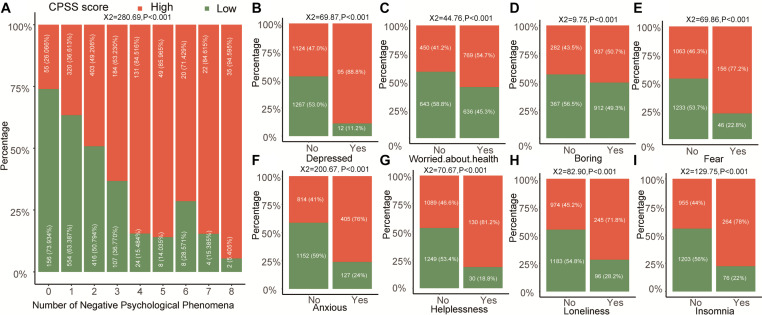
The relationship between CPSS score with different negative psychological phenomena in medical students. **(A)** The trend of the number of negative psychological phenomena in high or low CPSS score groups. **(B–I)** The individual negative psychological phenomenon.

In general, stress and other emotions interacted in an individual person, and we still detected the relationship of the positive and negative effects of psychologies with CPSS score in our study. With the increasing number of positive psychologies, the frequency of high CPSS score is less and less in the medical students (Figure 4A, *P* < 0.001). Separately, we found that concern for other people (Figure 4C), keeping protection from COVID-19 (Figure 4D), and keeping good health (Figure 4F) could significantly reduce the CPSS score (*P* < 0.001) in the medical students, but not for unlike public morality (Figure 4B, *P* = 0.07) and following the epidemic news (Figure 4E, *P* = 0.36). Unlike positive phenomena, the negative phenomena of medical students including anxiety, depression, worried about health, boring, fear, helplessness, loneliness, and insomnia in the high CPSS score group were all higher than that in the low CPSS score group (Figures 5A–I, all *P* < 0.001).

**FIGURE 6:**
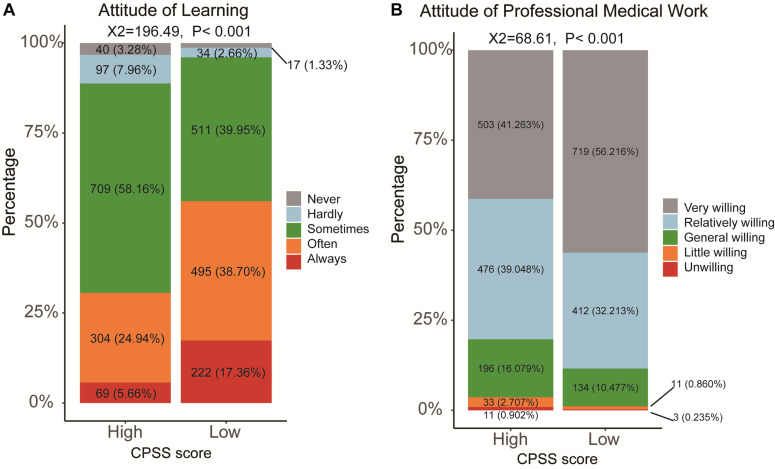
The changing of the attitudes of learning and professional work in medical students with high or low CPSS score. **(A)** Learning; **(B)** the professional work (medical work).

We also aimed to detect the relationship between CPSS and the attitude of learning and professional medical career. Then, learning and professional medical career attitudes with five levels were filled in by the subjects of the questionnaire. We could find that a high CPSS score led to the low enthusiasm for learning among medical students (Figure 6A, *X*^2^ = 196.49, *P* < 0.001). A five-level category for the attitude of professional medical career, including very, relatively, general, slightly willing, and unwilling, was further analyzed (Figure 6B). Although most of the medical students in the high- and the low-CPSS score groups (80.26 and 88.42%, respectively) continued their medical career, a high CPSS score still could cause a high proportion of students to be slightly willing and unwilling to do medicine-related work in the future (Figure 6B, *X*^2^ = 68.61, *P* < 0.001). To better comprehend the differences in the professional medical career attitude in the high- and the low-CPSS score groups in medical students, we also required the subjects to support the diversity of reasons for being unwilling to select the medical career in the questionnaire. Then, we divided these reasons into two parts, good and bad. The good reasons, such as “meaningful work” and “devotion of love,” were significantly higher in the medical student with low CPSS score. However, the poor reasons, such as “Disrespect by Patients,” “low salary,” “hard work and serving people,” “high academic requirements,” “Non-conformity for Career Planning,” and “Career without Future” were all higher in the medical students with high CPSS score (Table 3, all *P* < 0.05).

**TABLE 3:** The differences of the diverse reasons for choosing the medical career in medical students with high or with low CPSS score.

**Stratified**	**Reasons for medical work**	**High CPSS score(*n* = 1,219)**	**Low CPSS score(*n* = 1,279)**	***X*^2^**	***P***
Good	Meaningful work	896(73.50%)	1,061(82.96%)	32.868	**<0.001**
	Devotion of love	590(48.40%)	736(57.54%)	20.957	**<0.001**
	Stable workplace	671(55.05%)	750(58.64%)	3.288	0.07
	Easy employment	444(36.42%)	499(39.01%)	1.784	0.182
Poor	Work at risk	882(72.35%)	918(71.77%)	0.104	0.747
	Disrespect by patients	556(45.61%)	496(38.78%)	11.946	**0.001**
	Low salary	465(38.15%)	320(25.02%)	49.904	**<0.001**
	Strict college entrance examination	410(33.63%)	385(30.10%)	3.59	0.058
	Hard work and serving people	407(33.39%)	328(25.65%)	18.02	**<0.001**
	High academic requirements	332(27.24%)	289(22.60%)	7.193	**0.007**
	Non-conformity for career planning	255(20.92%)	190(14.86%)	15.673	**<0.001**
	Career without future	145(11.89%)	94(7.35%)	14.904	**<0.001**

*Chi-square test for categorical variables; Bold font means there is a significant difference between the two groups.*

## Discussion

As we know, COVID-19 is highly contagious with 20% severe illness and 2% mortality rate. In such a short period, the sharply increasing number of COVID-19-infected people may lead to students who are suffering from psychological strain of disease outbreak, anxiety, and other disruptive emotions. As we can see in most cities in China, the government had to shut down schools and some entertainment or gathering place at all levels and did not allow students to participate in various forms of social activities and entertainment for nearly 2 months. “Homestay” was really against normal learning tools but was necessary in the pandemic period. Importantly, we can easily clarify the stress situations of college students by using the CPSS tool, which is a brief assessment of someone’s stress level in any aspect of life situation (Cohen et al., 1983). Considering that medical students suffer a huge amount of stress from academics and employment, they deeply need to learn medicine knowledge in the available online courses. Thus, these reasons would raise the levels of their stress during the pandemic, especially for medical students with their extensive duties (Arora, 2015). Of note, mild, moderate, and high levels of stress and even burnout have been reported among medical students and healthcare professionals in other countries (Al Khalidi and Wazaify, 2013; Muzafar et al., 2015; Aamir et al., 2017; Alkot et al., 2017; Syed et al., 2019). In total, understanding of the distribution and reasons of high stress in students during the COVID-19 outbreak may be helpful to governments, schools, or health authorities.

This study firstly evaluated the distribution and huge differences of stress levels between medical and non-medical students in almost 31 provinces of China. To demonstrate the source of the high stress in medical students, we obtained four influencing factors, which could be significantly related to the progression of CPSS score in medical students by using the univariate and multivariate analysis, such as major of students, status of the intern student, family income, and level of familiarity with COVID-19. By comparing the female and older students with the male or young students, it was indicated that they have more mental problems, which is similar to the previous study (Cao et al., 2020; Li H. Y. et al., 2020; Liu C. Y. et al., 2020); however, statistical analysis showed no significant difference. In our study, approximately 85% of them were female medical students who could respond to the results. Among the population who answered the questionnaire, 66% of the students are nursing majors. Obviously, compared with other clinical majors, nursing students would face higher stress when they are undergoing the strictly learning and work environments (Magnavita and Chiorri, 2018). Additionally, medical interns have higher stress than the non-interns medical students (Babar et al., 2004; Liselotte et al., 2006; Guo et al., 2019; Shadid et al., 2020). Not only the clinical practice and emergencies in hospital, but also the task of publishing a graduation dissertation sends these intern students into a high-stress situation. What’s more, medical intern students need to avoid making mistakes because of the importance of patients’ life, because during the throat swab collection and medical operations, safety is uncertain all the time. Thus, responsible teachers and hospital managers should attend to these students, especially the nursing interns, regardless of school and hospital. It’s easy to understand that there is another reason that low family income tends to make students feel inferior, resulting in high stress in most instances (Wang et al., 2019). Finally, it is exceedingly profound to know that the higher level of familiarity with COVID-19 can lessen the anxiety and depression level of these students (Huang et al., 2020), as well as the stress in our study. People becoming familiar with things will significantly reduce psychological stress, fear, and other pessimistic moods. Therefore, it becomes much more important to reduce psychological stress through the comprehensive and accurate education of medical students in the prevention and control of the COVID-19 pandemic.

The survey also asked students to fill other positive and negative psychological emotions in the form voluntarily in the study. The negative psychological emotions included depression, worry, boredom, fear, anxiety, helplessness, loneliness, and insomnia, and these were all significantly related to the higher CPSS score in the medical students. Previous studies have reported that stress is closely related to negative psychology that can lead adolescent students to avoid coping, and avoidance of coping enhances the severity of psychological stress (Ozawa, 2010; Arsenio and Loria, 2014). Positive psychological emotions including five psychological aspects, namely, “health protection measures,” “following the epidemic news,” “health condition,” “concern about public morality,” and “caring for others” were also related to psychological stress. It is obvious that the positive states of students are lower under the high pressure in the pandemic period. Therefore, it is equally important to find other psychological changes to improve the status of the students’ physical and mental health.

The attitudes of learning and professional medical career were also reduced by the high stress brought by the epidemic. For the attitude of learning, medical students with low CPSS scores are more comfortable with learning than those with high CPSS scores. Researches showed that the lower psychological stress for learners who take the initiative to study in professional courses or read extracurricular books in online learning, the more efficient of the studying (Zhang et al., 2020). We also proved that most of the medical students both in the high- and the low-CPSS score groups would continue their medical careers. However, a higher proportion of medical students would be slightly willing and unwilling to do work related to medicine in the medical students with high stress. The medical students with low CPSS scores had a more stable professional mentality that was extremely helpful in controlling their occupational risks, and their motivation reasons are undeniable. In the pandemic period, for instance, from the arrival of the first medical team in Wuhan on New Year’s Eve on March 1 (Li Z. et al., 2020), a total of 42,322 medical staff used their spirit of selflessness and careers professionally, and this affected these medical students to reduce their stress. After COVID-19 in China, medical students would enhance their sense of social responsibility and professional attitude awareness. Thus, we strongly recommend that experts address these problems to improve the attitudes of learning and professional work by completing the formulation of sound incentive schemes.

### Limitations

This cross-sectional study had certain limitations. Although we have collected much demographic information and made a lot of data analysis, we cannot determine the causal relationship between stress and these indicators, the same as the relationship between positive psychology and negative psychology and stress. Further longitudinal research is needed to obtain the final causality and improve decision-making ability. In addition, data was collected by using self-administered questionnaires/instruments. Hence, we cannot rule out information bias. To enhance the applicability of the research results, we should further expand the sample size and improve the representativeness in the follow-up research. Despite these limitations, this study provided invaluable information related to the students during the COVID-19 outbreak across 31 provinces and autonomous regions in China, and our results can be used as a historical reference.

## Conclusion

Our study showed that the distribution of psychological stress (CPSS score) of the college students was obviously different among the different provinces in China during the outbreak of COVID-19. Among them, medical students suffer from higher stress than non-medical students in total and in most of the provinces. The top four independent risk factors related with psychological stress, including the lower level of familiarity with COVID-19, older age, lower family income, and the intern student, could significantly increase the psychological stress in the medical students in the pandemic period. Meanwhile, stress was related to some common positive and all the negative psychological phenomena significantly. Finally, timely decreasing of medical students’ stress can correct their learning attitudes and establish positive professional attitudes in the outbreak of COVID-19. The findings of the present study mainly could arouse the concern of policymakers, especially in the department of governments, schools, or health authorities.

## Data Availability Statement

The raw data supporting the conclusions of this article will be made available by the authors, without undue reservation, to any qualified researcher.

## Ethics Statement

The studies involving human participants were reviewed and approved by the Medical Ethics Committee of Xiamen Medical College. The patients/participants provided their written informed consent to participate in this study.

## Author Contributions

WY and XY conceived, designed, and carried out the study and prepared the first draft of the manuscript. YL helped design and carry out the study and prepared the first draft of the manuscript. QL assisted in design, data extraction, and interpretation of results. SV helped design and revised this manuscript. XY and YG performed data analysis. XY, HY, YZ, and CZ critically evaluated earlier drafts of the manuscript. All authors contributed to the article and approved the submitted version.

## Conflict of Interest

The authors declare that the research was conducted in the absence of any commercial or financial relationships that could be construed as a potential conflict of interest.
