# Supplementary material for: Effect of the Novel Coronavirus Pneumonia Pandemic on Medical Students’ Psychological Stress and Its Influencing Factors
Source: Front Psychol. 2020 Oct 14;11:548506. doi: 10.3389/fpsyg.2020.548506 (PMC7591817; doi:10.3389/fpsyg.2020.548506)
Supplement: Supplementary file 1 [file Data_Sheet_1.pdf]

Supplemental FigureS1: the design flowchart of present study

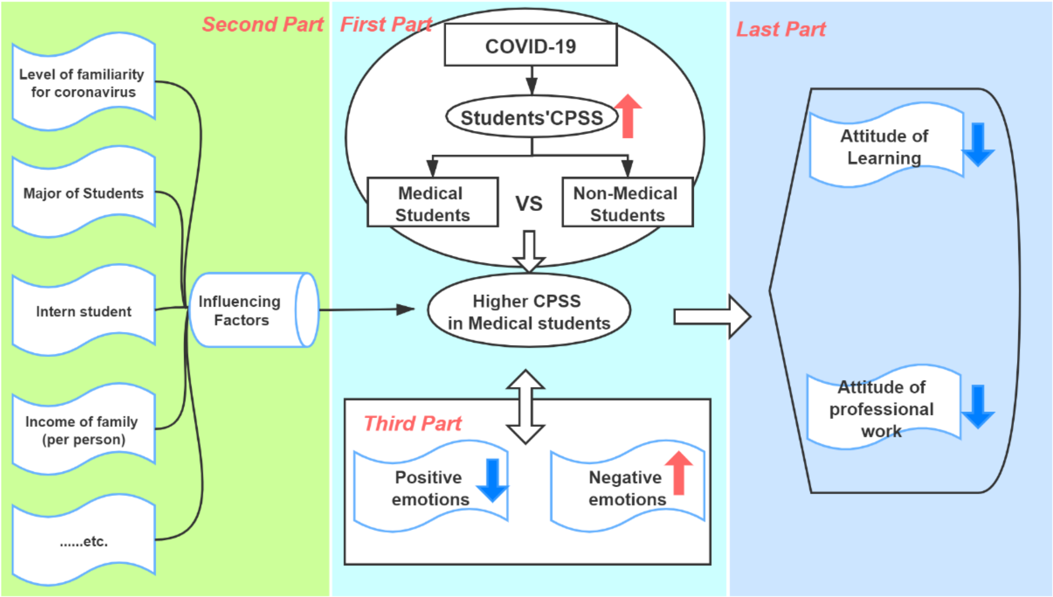

Figure legend: The design flowchart of present study. First, the differences of CPSS score between medical and non-medical students. Second, the influencing factors significantly related to high CPSS score. Third, the relationship of stress with negative psychologies and positive psychologies in the medical students. Last, the influences of high CPSS scores on medical students on the attitudes of learning and professional work.

Supplemental TableS1: The distribution of CPSS in each province of China for the college students during the novel coronavirus pneumonia pandemic

	All students			Medical students		
Provinces	CPSS score	SD	Number	CPSS score	SD	Number
Anhui	21.490	7.6489	49	24.208	6.4470	24
Beijing	25.750	4.3493	4	26.333	5.1316	3
Fujian	23.238	6.9831	2361	23.700	6.7638	1517
Gansu	24.065	5.9068	124	24.259	5.9740	112
Guangdong	23.098	8.2250	51	22.556	7.5056	27
Guangxi	23.500	6.8087	40	23.905	6.8986	21
Guizhou	24.855	6.2221	69	25.518	5.5759	56
Hainan	26.105	4.0946	19	25.636	4.2725	11
Hebei	25.073	6.7630	437	25.086	6.8599	348
Henan	21.436	5.8902	55	22.333	6.2670	24
Heilongjiang	27.143	7.5151	7	25.500	6.7157	6
Hubei	26.455	6.4554	11	21.800	6.9065	5
Hunan	24.478	7.5189	23	25.438	7.5716	16
Jilin	18.400	11.1490	5	18.333	12.5831	3
Jiangsu	23.967	7.3686	92	23.989	7.4253	90
Jiangxi	22.511	5.9682	45	24.105	4.7010	19
Liaoning	23.222	9.6667	9	20.750	6.6279	8
Neimenggu	20.333	.5774	3	20.333	.5774	3
Ningxia	21.800	5.4955	5	21.000	7.5498	3
Qinghai	27.571	5.0796	14	27.308	5.1863	13
Shandong	26.769	6.7596	13	27.889	7.0257	9
Shanxi#	24.018	6.5870	55	24.224	6.5328	49
Shaanxi#	23.800	7.3881	25	26.333	6.6583	3
Shanghai	27.333	1.5275	3	26.000		1
Sichuan	25.644	8.3996	45	27.226	8.1023	31
Tianjin	23.512	6.8159	41	23.667	6.9484	39
Xizang	27.400	2.3022	5	27.400	2.3022	5
Xinjiang	26.400	2.8810	5	26.400	2.8810	5
Yunan	26.750	8.6288	24	26.696	8.8185	23
Zhejiang	27.800	11.6570	15	30.100	12.7667	10
Chongqing	26.238	7.0349	21	28.214	6.8520	14
Total	23.655	6.9999	3675	24.137	6.8251	2498

Shanxi or Shaanxi were the two different pronunciations in two provinces in China
